# The role of Cra in regulating acetate excretion and osmotic tolerance in *E. coli *K-12 and *E. coli *B at high density growth

**DOI:** 10.1186/1475-2859-10-52

**Published:** 2011-06-30

**Authors:** Young-Jin Son, Je-Nie Phue, Loc B Trinh, Sang Jun Lee, Joseph Shiloach

**Affiliations:** 1 Biotechnology Core Laboratory, NIDDK, NIH, Bldg 14A Room 173, Bethesda, Maryland, 20892, USA; 2 Industrial Biotechnology and Bioenergy Research Center, KRIBB, Daejeon, 305-806, Korea

## Abstract

**Background:**

*E. coli *B (BL21), unlike *E.coli *K-12 (JM109) is insensitive to glucose concentration and, therefore, grows faster and produces less acetate than *E. coli *K-12, especially when growing to high cell densities at high glucose concentration. By performing genomic analysis, it was demonstrated that the cause of this difference in sensitivity to the glucose concentration is the result of the differences in the central carbon metabolism activity. We hypothesized that the global transcription regulator Cra (FruR) is constitutively expressed in *E. coli *B and may be responsible for the different behaviour of the two strains. To investigate this possibility and better understand the function of Cra in the two strains, *cra *- negative *E. coli *B (BL21) and *E. coli *K-12 (JM109) were prepared and their growth behaviour and gene expression at high glucose were evaluated using microarray and real-time PCR.

**Results:**

The deletion of the *cra *gene in *E. coli *B (BL21) minimally affected the growth and maximal acetate accumulation, while the deletion of the same gene in *E.coli *K-12 (JM109) caused the cells to stop growing as soon as acetate concentration reached 6.6 g/L and the media conductivity reached 21 mS/cm. *ppsA *(gluconeogenesis gene), *aceBA *(the glyoxylate shunt genes) and *poxB *(the acetate producing gene) were down-regulated in both strains, while *acs *(acetate uptake gene) was down-regulated only in *E.coli *B (BL21). These transcriptional differences had little effect on acetate and pyruvate production. Additionally, it was found that the lower growth of *E. coli *K-12 (JM109) strain was the result of transcription inhibition of the osmoprotectant producing *bet *operon (*betABT*).

**Conclusions:**

The transcriptional changes caused by the deletion of *cra *gene did not affect the activity of the central carbon metabolism, suggesting that Cra does not act alone; rather it interacts with other pleiotropic regulators to create a network of metabolic effects. An unexpected outcome of this work is the finding that *cra *deletion caused transcription inhibition of the *bet *operon in *E. coli *K-12 (JM109) but did not affect this operon transcription in *E. coli *B (BL21). This property, together with the insensitivity to high glucose concentrations, makes this the *E. coli *B (BL21) strain more resistant to environmental changes.

## Background

Acetate accumulation is one of the main concerns during high cell density growth of *E. coli *[1,2]. It was established that acetate concentrations above 40 mM (2.4 g/L) negatively affect cellular growth and recombinant protein production [3-5]. Acetate accumulation is dependent on the bacterial strain [6] and is affected by high growth rate and low oxygen concentration [4,7]. Methods have been developed to reduce acetate accumulation, including different glucose feeding strategies, usage of lower acetate producing carbon sources, and the development of mutant strains with altered acetic acid metabolic flux [8-10].

The acetic acid production pattern of *E. coli B *(BL21) is different from that of *E. coli *K-12 (JM109) especially when the bacteria grow to high densities at high glucose concentrations [11]. *E. coli *K-12 (JM109) accumulates acetate up to 11 g/L and its growth rate slows down; *E. coli *B (BL21) on the other hand, accumulates acetate to about 3 g/L and its growth rate is not affected. Careful evaluation of these two strains revealed that *E. coli *B (BL21) has active glyoxylate shunt, gluconeogenesis, anaplerotic pathway, and TCA cycle compared with *E. coli *K-12 (JM 109) [12,13]. It seems that in *E. coli *B (BL21), the central carbon metabolism pathways associated with glucose consumption are operating at the same rate regardless of the glucose concentration. Based on the above finding, it was suggested that FruR is responsible for the difference in the glucose metabolism of these two *E. coli *strains.

FruR, also known as, Cra (Catabolic repressor/activator), is a global transcription regulatory protein in enteric bacteria that regulates gene expression by binding to a specific DNA sequence [14]. It was reported that the *fruR *gene modulates the direction of carbon flow in *E. coli *by transcriptional activation of genes that encode enzymes associated with oxidative and gluconeogenic carbon flow and by repression of genes that are associated with fermentative carbon flow [15,16]. Cra is a common activator of the gene set *ppsA*, *fbp*, *pckA*, *aceA*, which are vital for acetate uptake. Sugar catabolites tend to bind to Cra and displace it from the operator sites in target operons [16]. These catabolites are present at high concentrations during growth in the presence of sugars, but in low concentration during growth in the presence of gluconeogenic substances. Genes that are activated by Cra (e.g. *ppsA, pckA*, and *aceBA*), are generally subject to catabolite repression, while genes that are repressed by Cra (e.g. *fru, pts, *and *edd*), are subjected to catabolite activation [16]. Most studies on the role of Cra in *E.coli *have used *E.coli *K-12 such as DH5α, BW25113, and MG1655 at low cell densities. The inactivation of Cra in plasmid-bearing *E. coli *DH5α altered metabolic gene expression, improved growth rate [17], and improved plasmid yield [18], while the deleting of *cra *gene had no distinct effect on the phenotype of *E. coli *BW25113 [19]. *cra *deletion in *E. coli *MG1655 contributed to accumulation of pyruvate by suppressing gluconeogenesis due to the decreased expression of *ppsA *and *pckA *[20].

Considering what is currently known about Cra regulation, and the difference between *E. coli *K-12 (JM109) and *E. coli *B (BL21) in regard to glucose metabolism, it was suggested that in *E. coli *B (BL21) *cra *is constitutively expressed, which explains why acetate is not accumulated. In the present work, we have investigated the effect of *cra *deletion on the central carbon metabolism in *E. coli *B (BL21) and *E. coli *K-12 (JM109) to verify the above assumption and to understand further the function of Cra in high cell density at high glucose, by following growth kinetics and gene expression.

## Results

### Cells growth parameters of the *cra*-positive and the *cra*-negative strains

**Figure 1:**
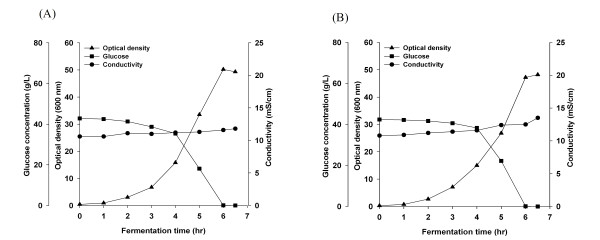
**Growth, glucose consumption, and conductivity of *E. coli *B (BL21)**. (A) *E. coli *B (BL21), (B) *E. coli *B (BL21) *cra*¯.

**Figure 2:**
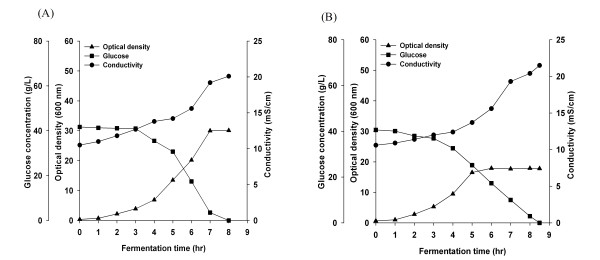
**Growth, glucose consumption, and conductivity of *E. coli *K-12 (JM109)**. (A) *E. coli *K-12 (JM109), (B) *E. coli *K-12 (JM109) *cra*¯.

**Figure 3:**
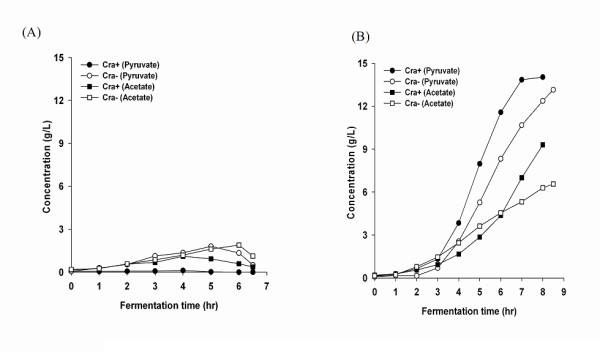
**Pyruvate and acetate production of *E. coli *B (BL21) and *E.coli *K-12 (JM109)**. (A) *E. coli *B (BL21), (B) *E. coli *K-12 (JM109).

Growth kinetics, glucose consumption and media conductivity profile of *E. coli *B (BL21) and *E. coli *K-12 (JM 109), without modification and with the *cra *gene deletion (*cra‾*), are shown in Figure 1 and 2, and acetate and pyurvate production kinetics are shown in Figure 3. *cra *deletion in *E. coli *B (BL21) minimally affected growth, final cell yield, glucose consumption and maximal acetate and pyruvate accumulation. Unlike *E. coli *B (BL21), *cra *deletion in *E. coli *K-12 (JM109) affected the cell growth pattern. The final OD_600 _was 30 in *E. coli *K-12 (JM109) and 18 in *E. coli *K-12 (JM109) *cra‾ *, maximal acetate and pyruvate concentrations in *E. coli *K-12 (JM109) were 9 g/L and 14 g/L respectively, and in *E. coli *K-12 (JM109) *cra‾ *acetate and pyruvate concentration reached 6.6 g/L and 13 g/L respectively.

Media conductivity values are shown in Figure 1 and 2. The conductivity increased from 10 mS/cm to 11 mS/cm in *E. coli *B (BL21) and from 10 mS/cm to 13 mS/cm in *E. coli *B (BL21) *cra‾*. In *E. coli *K-12 (JM 109), the conductivity reached 20 mS/cm and in *E. coli *K-12 (JM109) *cra‾*, reached 21 mS/cm.

### Effect of salt concentration on cell growth

To evaluate the effect of the media conductivity (salt concentration) on the bacterial growth, *E. coli *B (BL21) with and without *cra*, and *E. coli *K-12 (JM 109) with and without *cra*, were grown in regular LB media containing 5 g/L (conductivity 10 mS/cm) and in LB media containing 15 g/L NaCl (conductivity 22 mS/cm).

**Figure 4:**
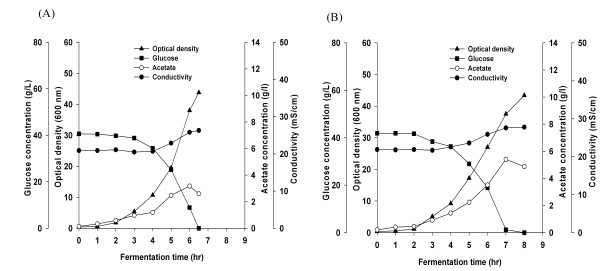
**Growth, glucose consumption, conductivity, and acetate production of *E. coli *B (BL21) in LB media containing 15 g/L NaCl**. (A) *E. coli *B (BL21), (B) *E. coli *B (BL21) *cra*¯.

**Figure 5:**
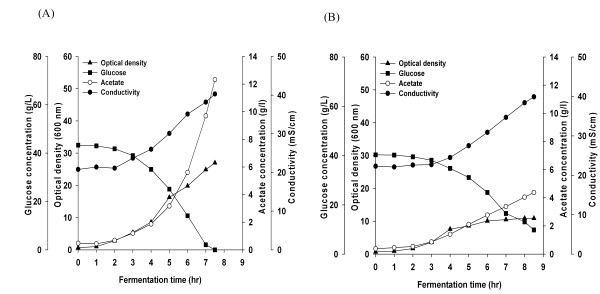
**Growth, glucose consumption, conductivity, and acetate production of *E. coli *K-12 (JM109) in LB media containing 15 g/L NaCl**. (A) *E. coli *K-12 (JM109), (B) *E. coli *K-12 (JM109) *cra*¯.

The growth of the different strains and media conductivity are shown in Figure 4 and 5. Both *E. coli *B (BL21) strains were not affected by the high conductivity media both strain grew to an OD_600 _of 44 and the media conductivity reached 26-27 mS/cm. However, there was a difference between the two *E. coli *K-12 strains; *E.coli *K-12 (JM109) grew to final OD_600 _of 30 in regular LB media and to 27 OD_600 _in the high conductivity media, while *E. coli *K-12 (JM109) *cra*‾ grew to final OD_600 _of 18 in the regular media and to 11 OD_600 _in the high conductivity media. During the growth of both *E. coli *K-12 strains, the media conductivity reached 40 mS/cm.

### Comparative transcription analysis by microarray and real time PCR

**Figure 6:**
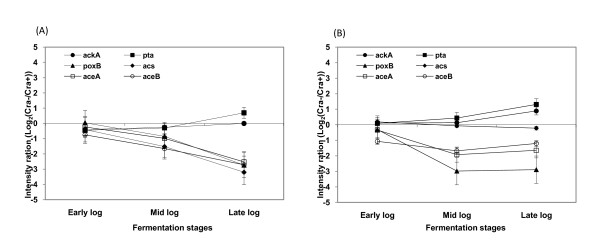
**Microarray analysis of glyoxylate cycle related genes and acetate utilization genes in *E. coli *B (BL21) and *E. coli *K-12 (JM109)**. (A) *E. coli *B (BL21), (B) *E. coli *K-12 (JM109) Abbreviations: *aceA*, isocitrate lyase; *aceB*, malate synthase A; *ackA*, acetate kinase; *pta*, phosphotransacetylase; *poxB*, pyruvate oxidase B; *acs*, acetyl-CoA synthetase

**Figure 7:**
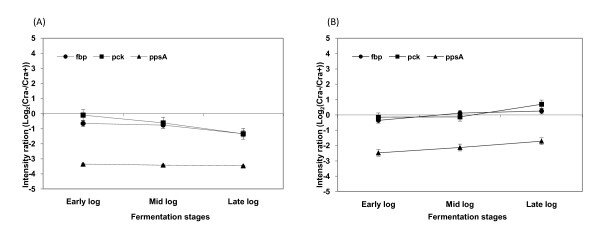
**Microarray analysis of glucoenogensis related genes in *E. coli *B (BL21) and *E. coli *K-12 (JM109)**. (A) *E. coli *B (BL21), (B) *E. coli *K-12 (JM109) Abbreviations: *fbp*, fructose-1,6-bisphosphatase; *pckA*, phosphoenolpyruvate carboxykinase; *ppsA*, phosphoenolpyruvate synthase

Results of microarray analysis, expressed as log 2 ratios of samples taken during the log phase growth from *cra *- negative strains and parental (*cra *- positive) stains, are shown in Figure 6 and 7. A positive ratio is an indication that gene transcription is higher in the *cra *-negative strains and negative ratio is an indication that the gene transcription is lower in the *cra *negative strain. The transcription comparison was done on the following pathways of the central carbon metabolism: the TCA cycle, the glyoxylate shunt, the gluconeogenesis, and the glycolysis. No significance transcription differences were identified in the glycolysis and the TCA cycle pathways (Data not shown) but differences were identified in the expressions of the glyoxylate shunt pathway genes (*aceA *and *aceB*), the acetate producing gene (*poxB*), and the gluconeogenesis gene (*ppsA*); all these genes were down regulated in both *cra‾ *strains while acetate uptake gene (*acs*) was down regulated significantly only in *E.coli *B (BL21) *cra‾*.

**Figure 8:**
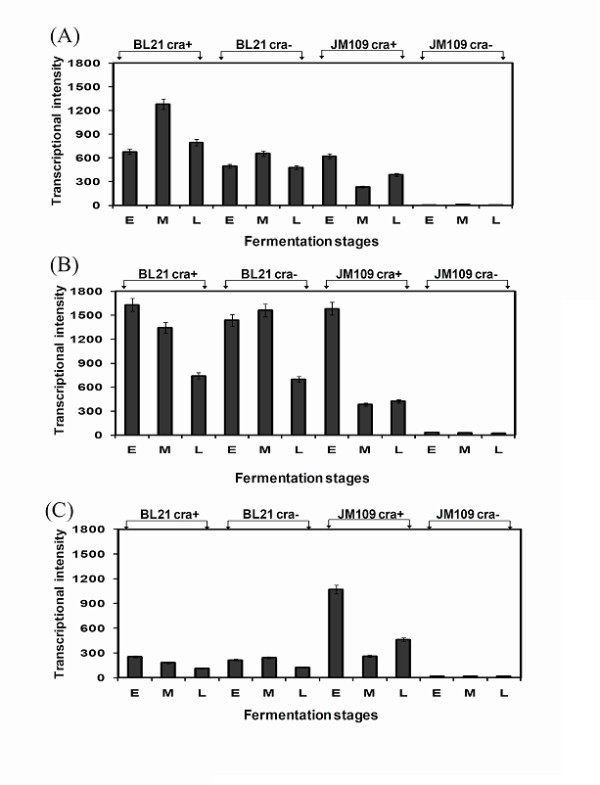
**Microarray analysis of *bet *operon genes in *E. coli *B (BL21) and *E. coli *K-12 (JM109)**. (A) *betT*, choline transporter, (B) *betA*, choline dehydrogenase, and (C) *betB*, betain aldehyde dehydrogenase.

The different response of the tested strains to high salt concentration (Figure 4 and 5) prompted us to look for transcription differences of the osmoprotectant *bet *operon genes (*betABT*): the results are shown in Figure 8. It is clear that the expression level of the *bet *operon genes (*betABT*) in *E. coli *K-12 (JM109) *cra‾ *are low compared with their expression in the *cra *- positive stain. This information was confirmed by Real Time PCR (Additional file 1, Figure S2, A, B, and C).

## Discussion

The purpose of this work was to find out if the global regulator Cra is responsible for the difference in glucose consumption and acetate production between *E. coli *K-12 (JM109) and *E. coli *B (BL21) as a result of its effect on the central carbon metabolism [21]. Based on published data about different growth parameters and metabolic activities of the two strains [11], we concluded that Cra may be constitutively expressed in *E. coli *B (BL21) but not in *E. coli *K-12 (JM109) [7]. By creating *cra *deleted mutants of these two strains and by analyzing their growth, metabolism and gene expression, we anticipated to have more information on the role of the Cra regulating the central carbon metabolism in *E. coli*, and perhaps be able to create a strain tolerant to high glucose concentrations. The results showed that *cra *deletion in *E.coli *B (BL21) minimally affected the growth and the maximal acetate accumulation, while in *E.coli *K-12 (JM109), *cra *deletion showed considerable effect. *E. coli *K-12 (JM109) grew to an OD of 30 and accumulated 9.0 g/l acetate, while *E. coli *K-12 (JM109) *cra‾ *stopped growing as soon as acetate concentration reached 6.6 g/L at an OD of 18; at this point, the media conductivity reached 21 mS/cm, indicating a possible effect on the ability of the mutant cell to keep its osmolarity.

Our microarray results showed that *cra *deletion affected the transcription of genes associated with acetate metabolism. *ppsA *(gluconeogenesis gene), *aceA *, *aceB *(the glyoxylate shunt pathway genes) and *poxB *(the acetate producing gene) were all down-regulated in both knockout strains, while *acs *(acetate uptake gene) was down regulated only in *E.coli *B (BL21) *cra*¯. These results are similar to previous reports that Cra represses transcription of genes that encode glycolytic (sugar-catabolizing) enzymes such as key enzymes in the Embden-Meyerhof and Enter-Doudoroff pathways, but activates transcription of genes that encode biosynthetic and oxidative enzymes such as key enzymes in the Krebs cycle, the glyoxylate shunt, the gluconeogenic pathway, and electron transfer [16]. Concerning the metabolic activity of the bacteria; the results presented here showed little or no effect of the *cra *deletion on acetate and pyruvate production. These observations are different from previous reports that Cra deletion in *E. coli *MG1655 contributed to accumulation of pyruvate by suppressing gluconeogenesis due to the down regulation of *ppsA *and *pckA *[20]. It is possible that Cra does not act alone, rather interacts with other pleiotropic regulators to create a network of metabolic effects [19].

Cra has been known to affect positively and negatively the transcription of several other genes such as *acnA/B *(aconitase), *icdA *(isocitrate dehydrogenase), *eda *(2-keto-3-deoxy-6-phosphogluconate adolase), *edd *(6-phosphogluconate dehydrase), *pfkA *(phosphofructokinase), and *pykF *(pyruvate kinase isoenzymes), but the transcription patterns of these genes in this work were not significantly different between the two strains (results not shown). Another global regulator that was reported to affect the glyoxylate shunt operon (*aceBAK*), is ArcA/B; this two-component system is considered to respond to oxygen limitation under microaerobic conditions [19]. In addition to Cra and ArcA/B, other metabolic regulators are involved in acetate metabolism in *E.coli*: acetyl-CoA synthetase is under the control of the sigma factors RpoS and RpoD and requires cAMP receptor protein, and the oxygen regulator (Fnr) for full expression [22]. Pyruvate oxydase B is also under the control of RpoS and cAMP-CRP. However, our work seems that *cra *deletion does not affect the transcription patterns of *cyaA*, *crp*, and *rpoD *in *E. coli *B (BL21) and *E. coli *K-12 (JM109) (Additional file 1, Figure S1, A and B). As for the transcription of *rpoS*, the level decreased significantly only at the late log phase in *E. coli *B (BL21) *cra‾ *but not in *E. coli *K-12 (JM109) *cra‾*. The down regulation of *rpoS *was reported to result in the high transcription of *poxB *and the low transcription of *acs *in *E. coli *B (BL21) [7].

An unexpected outcome of our work is the discovery that the *bet *operon, which has a role in protecting *E. coli *against high osmotic pressure [23-26], was down-regulated in *E. coli *K -12 (JM109) *cra¯ *strain. This phenomenon can provide a possible explanation to the growth inhibition of this strain when the media conductivity was above 18 mS/cm. The *bet *operon consists of three genes: *betT *(choline transporter), *betA *(choline dehydrogenase), and *betB *(betaine aldehyde dehydrogenase) [27]. The Choline transporter has high affinity for choline and transports choline into cell; choline dehydrogenase is an oxygen-dependent enzyme that converts choline into betaine aldehyde; and betaine aldehyde dehydrogenase converts betaine aldehyde into glycinebetaine [28]. The *bet *genes are induced by choline, oxygen, and osmotic stress [29]. The inhibition of cell growth that was observed only in *E. coli *K-12 (JM109) *cra ¯ *is likely the result of the limited transcription of the *bet *operon (Figure 8). The *cra *deletion affected the *bet *operon expression only in *E. coli *K-12 (JM109) and not in *E. coli *B (BL21); it is possible that Cra affects the bet operon indirectly. This is an additional difference between these two strains that may point to less regulation in *E. coli *B. The insensitivity of *E. coli *B (BL21) to the *cra *deletion, together with its insensitivity to high glucose concentration, enables this strain to be less affected by environmental stress such as glucose concentration and ionic strength. This may be the results of lacking an internal control mechanism that may be responsible for *E. coli *K-12 inability to cope with environmental stress.

## Conclusions

To evaluate the role of the global regulator Cra in the central carbon metabolism of *E. coli *K and *E. coli *B, the *cra *gene was deleted and the mutant strains were compared to the parental strains in their gene expression, growth and metabolic behaviour. The transcriptional changes caused by the deletion of *cra *gene support the assumption that Cra is a major regulatory molecule that has an effect on the expression of *ppsA*, a*ceBAK *and *acs *in *E.coli *B (BL21), but these transcriptional changes did not affect the activity of the central carbon metabolism, suggesting that Cra does not act alone, rather it interacts with other pleiotropic regulators to create a network of metabolic effects. An unexpected outcome of this work is the finding that *cra *deletion in *E. coli *K-12 (JM109) caused transcription inhibition of the *bet *operon which is responsible for maintaining the salt concentration in the cells. In comparison, the *bet *operon expression was not affected in *E. coli *B (BL21). This property, together with this strain insensitivity to high glucose concentrations, makes this strain more resistant to environmental changes and may be one of the reasons for the growth properties of this strain.

## Materials and methods

### Bacterial strains

*E. coli *B (BL21) (λDE3) (F^-^, *ompT, hsdSB (rB-, mB+), dcm, gal, Cmr*) and *E. coli *K-12 (JM109) (λDE3) (*endA1, recA1, gyrA96, thi, hsdR17 (rk-, mk+), relA1, supE44, Δλ-, Δ(lac-proAB), *F'*, traD36, proAB, lacIqZΔM15*) were used. Both strains were obtained from Promega Corp. (Madison, WI).

*cra *gene knock-out was carried out by recombination using plasmid pKD46 as previously described [30]. A kanamycin marker in plasmid pACYC177 (New England Biolab, Inc., Beverly, MA) was amplified by PCR to disrupt *cra *gene using two primers 

5'-GTGAAACTGGATGAAATCGCTCGGCT

GGCGGGAGTGTCGCGGACCACTGCTACGGTCTGCGTTGTCGGGAAGATGCG 

and

 5'-CTTAGCTACGGCTGAGCACGCCGCGGCGATAGAGATTACGTTTAATGCGC

GTACAAAGCCGCCGTCCCGTCAAGTCA.

 PCR products carrying kanamycin marker and homologous region (50 bp) were electroporated into JM109 (DE3) harboring pKD46 where lambda recombinase was fully expressed by L-arabinose during culture at 30°C, and subsequently cells were spread on LB agar containing kanamycin. Candidate colonies were grown at 42°C to cure plasmid pKD46 carrying the temperature sensitive origin of replication. Specific gene knock-out in JM109(DE3) *cra*¯strain was confirmed by PCR with primers which can hybridize upstream and downstream of deleted *cra *genes. The PCR products were also electroporated into MG1655 harboring pKD46 to make MG1655 *cra*¯strain. P1 lysates of MG1655 *cra*¯were used to transduce *cra *mutation carrying a kanamycin marker into BL21(DE3) strain to make BL21(DE3) *cra*¯ which were confirmed as described above.

### Fermentation and sample preparation

The four strains were grown at 37°C in modified LB medium containing 10 g/L tryptone, 5 g/L yeast extract (15 g/L for JM109), 5 g/L NaCl, and 5 g/L K_2_HPO_4_. After sterilization, 10 mM MgSO_4_, 1 ml/L trace metal solution [31], and 40 g/L glucose were added. Overnight cultures grown at 37°C were used to inoculate 3.0 L of medium in a B. Braun fermentor equipped with data acquisition and a control system. The cultures were grown to high-cell-density, the pH was controlled at 7.0 by the addition of 50% NH_4_OH, and dissolved oxygen was kept at 30% air saturation. For high-conductivity media, the NaCl was adjusted to 15 g/L, the other compositions were the same. Samples for acetic acid, pyruvate, glucose concentration, and conductivity analysis were collected at regular intervals, centrifuged at 14,000 × g for 5 min and the supernatant was analyzed with HPLC. Samples for total RNA purification were collected and centrifuged at 14,000 × g for 1 min at 4°C, the supernatant was removed, and the cell pellets were quickly frozen in dry ice and stored at -80°C.

### Analytical methods

The concentrations of acetate and pyruvate were analyzed at 35°C (for acetate) and 65°C (for pyruvate) on an HP 1100 series (Agilent, Santa Clara, CA) using an Aminex HPX-87H column (300 × 7.8 mm; Bio-Rad, CA, USA). The mobile phase was 0.008 N sulfuric acid and flow rate was 0.6 mL/min. After injection of a 20-μL sample, absorbance was monitored at a wavelength of 210 nm using a UV detector. Glucose in the culture supernatant was determined using an YSI glucose analyzer (YSI Inc., Yellow Springs, OH). Conductivity was analyzed at 30°C using CDM210 conductivity meter (Radiometer Analytical, Lyon, France).

### Total RNA preparation and microarrays

Total RNA was isolated using a MasterPure RNA Purification Kit (Epicentre Technologies, Madison, WI) and RNeasy Mini Kit (Qiagen, Valencia, CA) according to the manufacturers' protocols (MCR 85102 & 74104). RNA concentration and purity were determined by measuring absorbance at 260 nm (A_260_) using a NanoDrop 1000 (Thermo Scientific, Waltham, MA). Three biological replicates of RNA samples from *E. coli *B (BL21) and *E. coli *K-12 (JM109) *cra *knockout strain and wildtype were hybridized to Affymetrix *E. coli *Genome 2.0 Array. DNA-chip Analyzer (dChip version 2006) was used to process the raw data generated at the NIDDK Genomics Laboratory.

### Real-time PCR

Reverse transcription (RT) was carried out with the High Capacity RNA-to-cDNA Kit using the manufacturer's protocol (Applied Biosystems). A 20-ul RT reaction mixture included 2 ug of total RNA, 10 ul of 2X RT buffer, 1 ul of 20X RT enzyme mix, and DEPC-treated water. Real-time PCRs were carried out on a 7900 HT Fast Real-Time PCR System (Applied Biosystems) with the Power SYBR Green PCR Master Mix (Applied Biosystems). The PCR mixtures were incubated at 95°C for 10 min to activate the AmpliTaq DNA polymerase, followed by 40 cycles of amplification (95°C for 15 sec; 60°C for 1 min). A final extension step was performed at 60°C for 10 min. Real-time PCR results were also analyzed using the 7900 HT SDS software (Applied Biosystems).

## Competing interests

The authors declare that they have no competing interests.

## Authors' contributions

YS performed experiments and analysis, literature search and writing the manuscript, JP hypothesised the idea, survey the literature, analyze results and writing the manuscript, LT performed fermentation experiments, SL genetic work, JS conceived the study participated in the designed and coordination and the writing of the manuscript. All authors read and approved the final manuscript.

## Supplementary Material

Additional file 1 **Supplemental Figure S1 and S2**. Microarray data (Supplemental Figure S1) and real time PCR data (Supplemental Figure S2). Click here for file
