# Supplementary material for: The role of Cra in regulating acetate excretion and osmotic tolerance in E. coli K-12 and E. coli B at high density growth
Source: Microb Cell Fact. 2011 Jun 30;10:52. doi: 10.1186/1475-2859-10-52 (PMC3146397; doi:10.1186/1475-2859-10-52)
Supplement: Additional file 1 — Supplemental Figure S1 and S2. Microarray data (Supplemental Figure S1) and real time PCR data (Supplemental Figure S2). [file 1475-2859-10-52-S1.PDF]

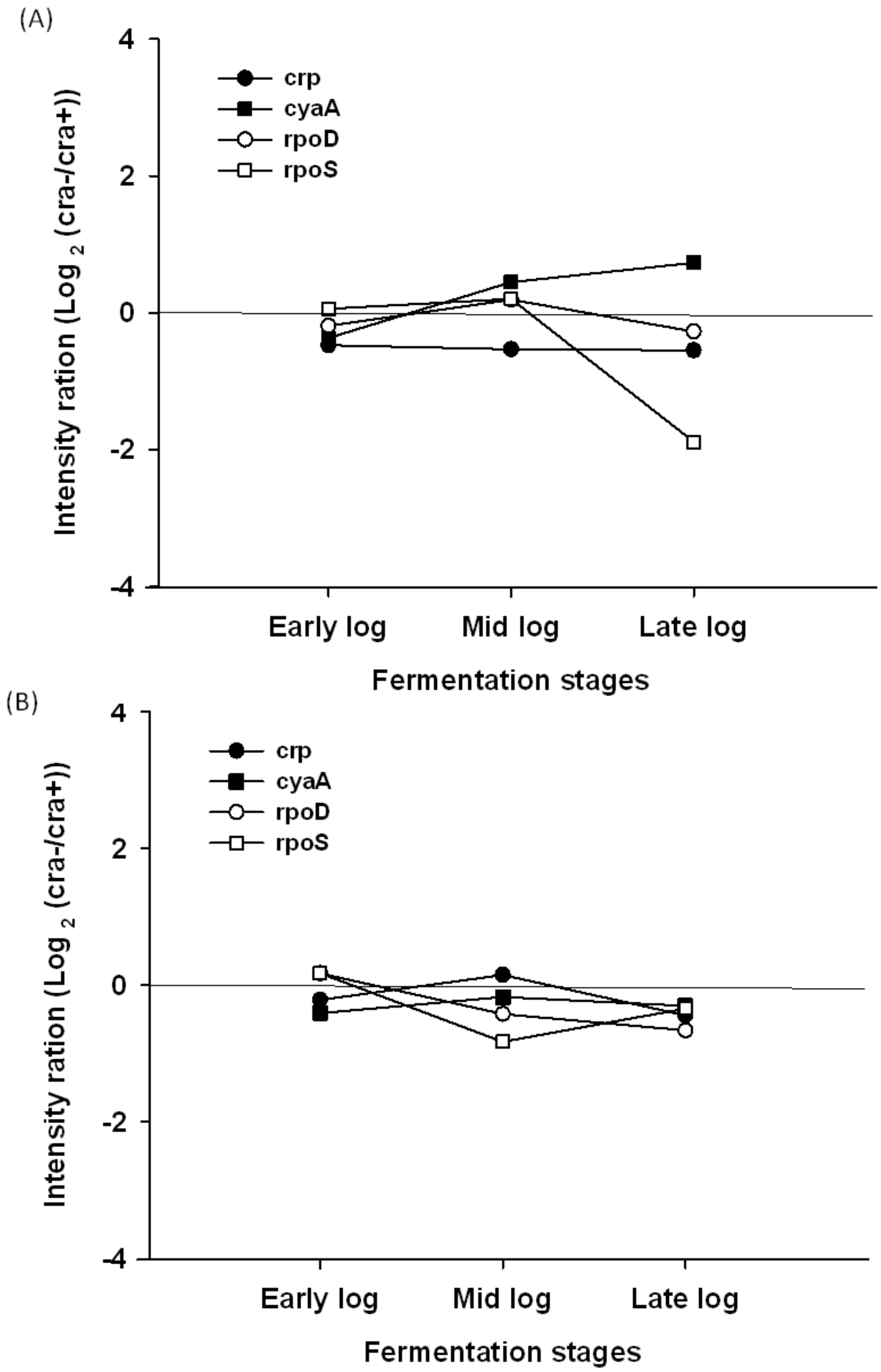

Supplemental Figure S1. Microarray analysis of selected regulatory genes.
(A) *E. coli* B (BL21), (B) *E. coli* K-12 (JM109) Abbreviations: *crp*, cAMP receptor protein; *cyaA*, adenylate cyclase; *rpoD* and *rpoS*, sigma factors

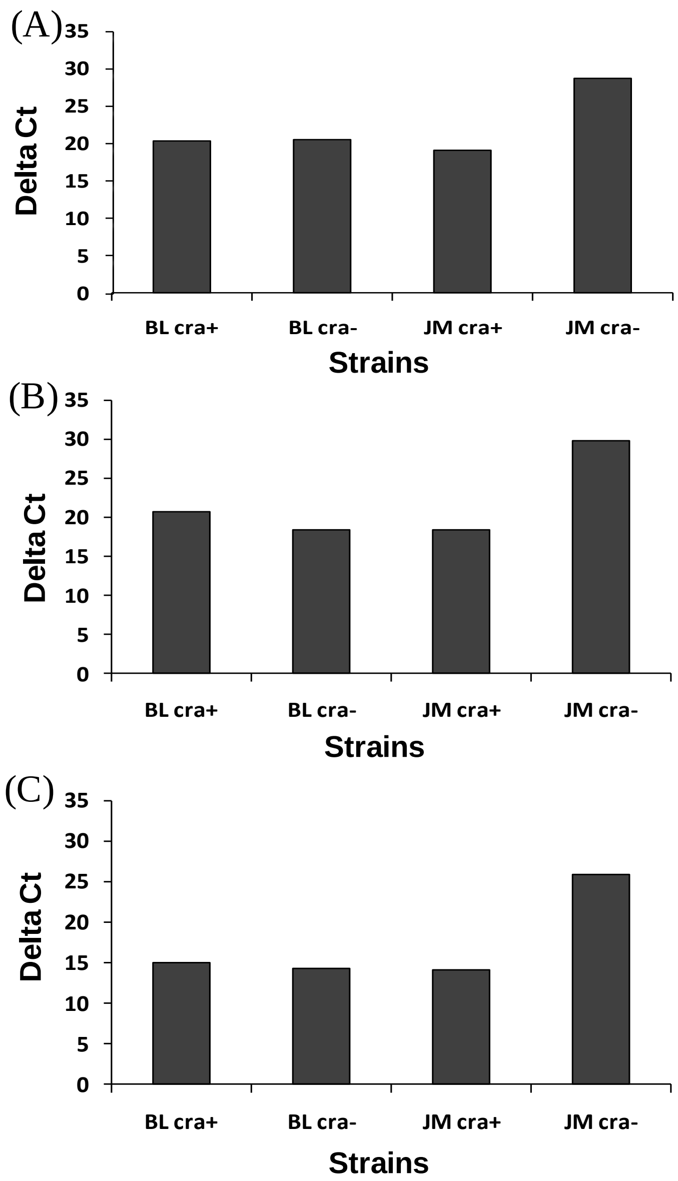

Supplemental Figure S2. Real-time PCR of *bet* operon genes in *E. coli* B (BL21) and *E. coli* K-12 (JM109).
(A) *betT*, choline transporter, (B) *betA*, choline dehydrogenase, and (C) *betB*, betain aldehyde dehydrogenase.
